# Optical Fiber Sensors Based on Polymeric Sensitive Coatings

**DOI:** 10.3390/polym10030280

**Published:** 2018-03-07

**Authors:** Pedro J. Rivero, Javier Goicoechea, Francisco J. Arregui

**Affiliations:** 1 Materials Engineering Laboratory, Department of Mechanical, Energy and Materials Engineering, Public University of Navarre, Campus Arrosadía S/N, 31006 Pamplona, Spain; 2 Nanostructured Optical Devices Laboratory, Department of Electrical and Electronic Engineering, Public University of Navarre, Campus Arrosadía S/N, 31006 Pamplona, Spain; javier.goico@unavarra.es (J.G.); parregui@unavarra.es (F.J.A.)

**Keywords:** sensors, optical fiber, polymers

## Abstract

Polymer technology is one of the fastest growing fields of contemporary research due to the possibility of using a wide variety of synthetic chemical routes for obtaining a polymeric network with a well-defined structure, resulting in materials with outstanding macroscopic properties. Surface engineering techniques based on the implementation of polymeric structures can be used as an interesting tool for the design of materials with functional properties. In this sense, the use of fabrication techniques for the design of nanostructured polymeric coatings is showing an important growth due to the intrinsic advantages of controlling the structure at a nanoscale level because physical, chemical, or optical properties can be considerably improved in comparison with the bulk materials. In addition, the presence of these sensitive polymeric coatings on optical fiber is a hot topic in the scientific community for its implementation in different market niches because a wide variety of parameters can be perfectly measured with a high selectivity, sensitivity, and fast response time. In this work, the two main roles that a polymeric sensitive matrix can play on an optical fiber for sensing applications are evaluated. In a first section, the polymers are used as a solid support for the immobilization of specific sensitive element, whereas in the second section the polymeric matrix is used as the chemical transducer itself. Additionally, potential applications of the optical fiber sensors in fields as diverse as biology, chemistry, engineering, environmental, industry or medicine will be presented in concordance with these two main roles of the polymeric sensitive matrices.

## 1. Introduction

Polymers are ubiquitous and currently they are present in any aspect of our lives: from the classic utilization as bags to preserve food, stationery material, protective cases, clothes, or paints, to the most recent uses in 3D printing or controlled drug-delivery applications. The fabrication and use of synthetic polymers has been a revolution that has led to the annual production of 300 million tons [1], which, for comparison, is similar to the world production of soybean. Among the countless applications of polymers, their utilization for the fabrication of sensors is very appealing [2]. Polymers are relatively low-cost materials, and they can be deposited in substrates made of different materials with diverse shapes and sizes. The continuous efforts and evolution in the methods to synthesize and analyze them has produced viable nanoprocessing techniques. Now, it is possible to create synthetic polymers in the forms of nanofibers or nanoparticles [3] to create mimics of polymers found in nature or even to synthesize biologically-inspired materials such as molecularly imprinted polymers (MIPs) with a capability of replacing their natural counterparts: antibodies or enzymes [4,5]. Since the properties of the polymers can be tuned, for instance, to achieve conducting films, there are a multitude of examples of polymers used in electronic sensors [6]. Not only electronic properties but also optical ones as well can be tailored. Even optical fibers have been fully fabricated with polymers [7,8].

Regarding this last technology, both plastic optical fiber [9,10,11] and, especially, fused silica optical fiber has meant an authentic revolution in telecommunications and in sensing fields in recent decades due to their intrinsic advantages (light-weight, portability, small size, remote sensing measurements or immunity to electromagnetic interferences) which are key factors for sensing or biosensing applications. In addition, the optical fiber field has shown considerable growth thanks to the implementation of sensitive coatings onto diverse optical fiber configurations. In this sense, the use of polymeric sensitive materials in optical fiber sensors has contributed to improving particular characteristics useful for sensing purposes such as an enhancement in the response time with a better sensitivity and selectivity [12,13,14,15].

**Figure 1:**
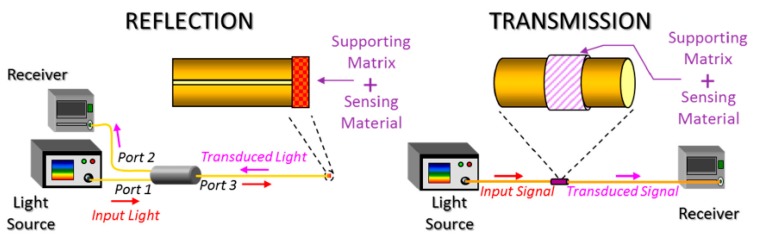
Schematic representation of two different architectures used for optical fiber sensors such as reflection mode (**left**) and transmission mode (**right**). Reprinted with permission of [16].

Another aspect related to optical fiber sensing is that depending on the location of the sensitive coating along the optical fiber, many different architectures can be found. For example, reflection or transmission optical setups can be considered as two basic configurations; see Figure 1. In the case of the reflection scheme, the sensing material can be deposited at the end of the fiber tip, and the light is guided from the source to the sensor, and from the sensor to the receiver by an optical fiber coupler. However, in the case of the transmission scheme, there is a direct optical path between the light source and the receiver, respectively, making possible different interactions between the guided light and the sensitive coating. In Figure 1, a representative scheme of both types of sensing configurations as a function of the location of the sensitive coating can be appreciated [16].

These two basic configurations can be used to exploit different optical phenomena such as optical absorbance, fluorescence, or evanescent field. They can also be enriched with additional coatings or elements to arrange different configuration such as the ones based on surface plasmon resonance (SPR) [17], lossy mode resonance (LMR) [18], fiber Bragg gratings (FBG) [19,20,21], long period gratings (LPG) [22] or photonic crystal fibers (PCF) [23], just to mention a few.

As mentioned, polymers are relevant in the fabrication of optical fiber sensors and this review is focused on the two main functions that a polymeric coating can play in the sensing configuration. In the first one, the polymeric matrix can be used as a solid support for the immobilization of a specific chemical transducer. In the second one, the polymeric matrix can be used as the chemical transducer itself [24]. Based on this classification, to have a basic perspective of this topic, some applications in fields as diverse as biology, chemistry, engineering, industry, or medicine will be briefly presented.

## 2. Polymers as a Support of the Sensitive Element

The aim of this section is to describe some works in which different chemical transducers such as metallic nanoparticles (gold, silver, copper), metal oxide nanoparticles (silica, titania, ceria), and luminescent materials (PtTFPP, Ruthenium complexes, quantum dots, colorimetric dyes) have been immobilized into a polymeric matrix. Polymer engineering allows an enormous flexibility for the design of innovative sensitive coatings and sensors, which are able to measure a wide variety of analytes of importance in the biomedical field, industrial processes, control of water quality or the monitoring of hazardous environments. 

### 2.1. Immobilization of Luminescent Materials

Photoluminescence is an optical phenomenon which consists of emission of light by a material as a consequence of its previous absorption at lower wavelengths (excitation). As a function of the lifetime of this emission, luminescence can be classified in two different modalities such as fluorescence (lifetime in the range of ps or ns) or well as phosphorescence (lifetime greater than ms). There are a wide variety of luminescent materials (organic dyes, quantum dots, porphyrins) which are entrapped in a specific matrix to promote an adequate interaction between the analyte and the sensing material, and also an efficient coupling of the emitted light to the optical fiber towards the detector. For the development of an adequate luminescent sensor, a good distribution of the luminescent material in this matrix is necessary to avoid an aggregation, causing self-quenching with a loss in the resultant sensitivity [25]. 

A good approach for the development of luminescent optical fiber sensors involves the entrapment of a suitable sensing reagent in a specific polymeric matrix, for instance by using Layer-by-Layer nano-assembly (LbL) [26,27,28,29]. In these works, luminescent optical fiber oxygen sensors have been fabricated by means of the LbL method by using the same oxygen-sensitive fluorophore, a metalloporphyrin known as platinum tetrakis pentrafluorophenyporphine (PtTFPP) which shows excellent features such as long lifetime emission, significant Stokes shift, photo, and thermal stability. Another aspect to point out regarding this reagent is that it is a water-insoluble complex, [26,27] the first bibliographic references being about the incorporation of PtTFPP into LbL films. The initial incompatibility between the sensing material and Layer-by-Layer can be overcome by using a PtTFPP emulsion, which would allow a further deposition of PtTFPP by this nanoassembly technique. In addition, this luminescent reagent shows an absorption peak at 390 nm, whereas the emission peak is centered at 650 nm, the resultant Stokes shift being 260 nm, which ensures no overlapping between both the excitation and the emission response signal.

**Figure 2:**
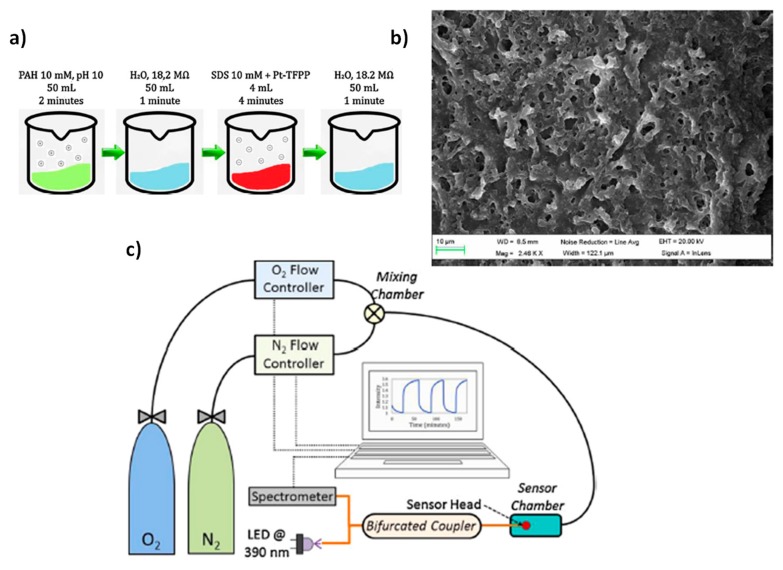
(**a**) Solutions required for the preparation of the Layer-by-Layer films as well as the duration of the immersion steps to construct a cycle; (**b**) SEM image obtained from an optical fiber once the construction process is over; (**c**) Experimental setup used to characterize the sensor. The flow controllers are electronically tuned by software. Reprinted with permission from Ref. [27].

In Figure 2, the experimental conditions for the preparation of the sensitive coating (Figure 2a) can be observed, which shows a good roughness (around 21 nm) and porosity (average size pore between 3 and 5 µm), which is one of the most important requirements for gaseous oxygen sensing (see Figure 2b). In addition, the resultant setup is presented in Figure 2c for the characterization of the sensor based on a reflection configuration. In this setup, it can be appreciated that the sensor is exposed to a constant gas flow, adjusting its composition with different oxygen and nitrogen proportions. The flow from each gas was mixed in a chamber and it was conducted through a tube, the fiber being located inside this tube and in close contact with the gas sensing.

**Figure 3:**
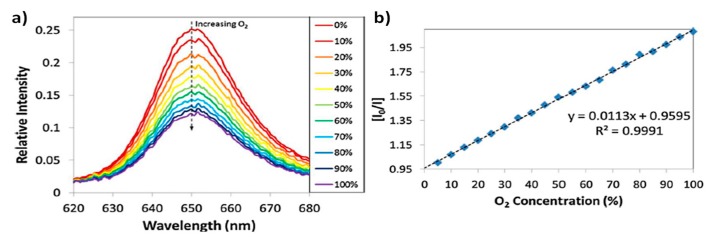
(**a**) Luminescence signal from the sensor when it is exposed to different increasing oxygen concentrations; (**b**) Stern-Volmer plot obtained from the sensor for increasing oxygen 5% steps. Inset the linear approach with the *R*^2^ parameter. Reprinted with permission from Ref. [27].

Figure 3 presents the sensor response to different oxygen concentrations from 0% to 100% in 10% steps. The experimental results indicate that the peak emission intensity is reduced as the oxygen concentration is gradually increased. The sensing mechanism relies on oxygen quenching. In other words, the luminescence is affected by molecular oxygen because there is a reaction with the excited state of the fluorophore, limiting the luminescence emission, so that the higher oxygen concentration, the lower emission is collected. In Figure 3a it can be clearly observed that when no oxygen is present (0%), the emission peak relative intensity is close to 0.25, whereas for a 100% of concentration, the emission peak falls to a value of 0.1182. It can be seen in the Stern-Volmer plot of Figure 3b that there is a linear relationship between the relative intensity of the emission and the resultant gas concentration [27]. 

**Figure 4:**
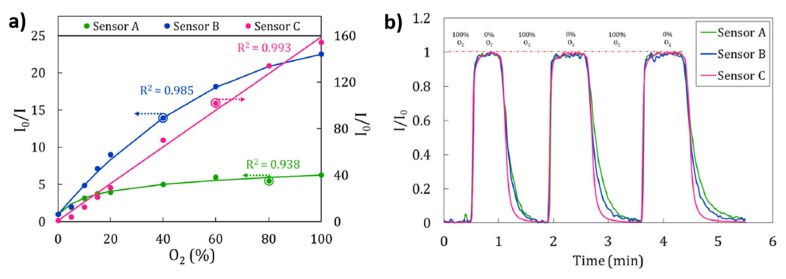
(**a**) Calibration curves of Sensors A (PDDA), B (PEI) and C (PAH). To make easier the comparison between the curves shapes, calibration curves of Sensors A and B are adjusted on the left axis, whereas that of Sensor C is adjusted on the right axis; (**b**) Response of the Sensors to dynamic variations of oxygen concentrations from 0% to 100% repeatedly in 3 cycles. Reprinted with permission from Ref. [28].

Other work based on the immobilization of PtTFPP by using Layer-by-Layer is focused on the influence of the polyelectrolytes of the polymeric matrix on the surface morphology [28]. In this work, three different polyelectrolytes such as poly(diallyldimethylammoniumchloride) (PDDA), polyethyleneimine (PEI) and poly(allylamine hydrochloride) (PAH) have been used for the entrapment of the luminescent material. Among them, the sensor composed of PAH (Sensor C) exhibited a higher sensitivity (*I*_0_/*I*_100_ = 154.35) to different oxygen concentrations in comparison with the sensor composed of PEI (Sensor B; *I*_0_/*I*_100_ = 22.54) or the sensor composed of PDDA (Sensor A; *I*_0_/*I*_100_ = 6.3), as it can be seen in Figure 4. An aspect related to this difference in sensitivity is associated to the properties of the polymeric matrix because both PDDA and PEI polyelectrolytes show similar morphological properties (a lower thickness and roughness than PAH structure). In addition, PDDA (Sensor A) shows a more hydrophilic nature in comparison with PEI (Sensor B), and due to this, Sensor A presents less permeability to gases, and in consequence, is expected to be less sensitive to oxygen. Finally, in terms of sensitivity, this Sensor C is similar to other sensors based on sol-gel matrices [30,31,32] and considerably better (up to 10 or 25 times) than those in which the luminescent material (PtTFPP) is embedded in other polymeric matrices [33,34]. Another aspect to take in consideration is the response and recovery times which are key factors for oxygen sensors. In this sense, among the three different sensors of study, the Sensor C showed a less time to recover (12 s) than for Sensors A and B (20.4 and 30 s, respectively), as it can be seen in Figure 4b. This difference in response time is a consequence of the morphology of the sensing coating because a rougher coating (PAH, sensor C) makes possible a faster recovery time to the maximum intensity in absence of oxygen. In other works [35], a study about the enhancement of luminescence for optical fiber oxygen sensors is presented as a function of the distance between the fluorophore layers, minimizing the self-quenching of fluorophores, by using different multilayer structures fabricated by the Layer-by-Layer technique.

**Figure 5:**
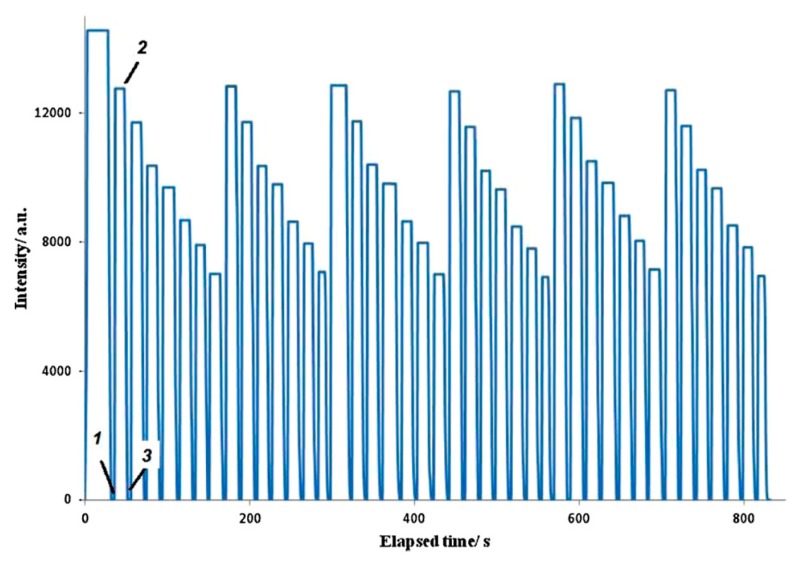
Steady-state fluorescence measurements over time (excitation 380 nm and emission 500 nm) of the dry optical fiber with six layers, followed by three cycles of Hg(II) aqueous solutions with in the following concentrations: 0, 0.01, 0.05, 0.1, 0.799, 1.99 and 2.69 μM. (1) The fiber was immersed in water, (2) removed from water, and (3) immersed in Hg(II) 0.01 μM. Reprinted with permission from Ref. [36].

According to these previous results, the use of Layer-by-Layer assembly is an alternative for an adequate immobilization of luminescent materials due to the high versatility and capability to adjust the resulting properties of the fabricated matrix in comparison with other deposition techniques. This aspect has been also corroborated in work [36] where the immobilization of carbon dot fluorescent nanomaterials on optical fiber is presented by using the Layer-by-Layer technique with the aim to detect Hg(II) ions. In this work, two key aspects for sensing applications have been demonstrated. The first one is an improvement of the sensor response time. The second one is an increase in the sensitivity because this LbL method shows a limit detection of 0.01 µM in comparison with the sol-gel method with a 0.1 µM [37]. Figure 5 represents the sensing coatings composed of 6 layers of PEI/fluorescent carbon dots immobilized by Layer-by-Layer where the reversible and reproducible behavior towards Hg(II) can be seen. In addition, an increase of number of layers from 1 to 6 has allowed a decrease in the detection limit from 0.1 µM (1 layer) up to 0.01 µM (6 layers) [36].

As previously mentioned, another aspect regarding the LbL technique is its high versatility to entrap diverse types of luminescent materials such as Ruthenium complexes for oxygen sensing applications [38,39]. In work [39] a study is performed regarding the number of bilayers (5, 50 and 125 bilayers, respectively) onto the end of the optical fiber with the aim of fabricating porous composite membranes with a good sensitivity. The experimental results indicated that 5-layer or 50-layers showed the best sensitivity and reproducibility. However, other matrices of sol-gel nature [40,41,42,43] or good organic polymeric matrices [44,45] can be also found in the bibliography for the immobilization of Ru complexes. Most of these chemical indicators respond to the excitation light by emitting a fluorescence signal near 650 nm (red color), showing a relatively large Stokes shift.

In work [40] the influence of sol-gel coated length and withdrawal rate on plastic optical fiber core is evaluated to obtain the best oxygen gas sensitivity. In work [41] the performance of dissolved oxygen optical fiber sensor in air and aqueous environment is investigated. In this work, it is demonstrated that the fluorescence intensity is higher for sensor in air than in aqueous environment, showing in both cases a decrease of the fluorescence intensity at higher oxygen concentrations. In work [42] the performance evaluation of optical fiber sensors is studied by using different oxygen sensitive nanomaterials which are coated on one end of optical fiber by using the same sol-gel process. The oxygen sensitive nanomaterials used are Pt (II) meso-tetra (pentafluorophenyl) porphine (PtTFPP), Tris (4,7-diphenyl-l,lO-phenanthroline) ruthenium (II) and Pd (II) meso-tetra (pentafluorophenyl) porphine (PdTFPP). The experimental results indicate that optical fiber sensor probe coated with PdTFPP exhibits the highest sensitivity while Ru (II) complex coated optical fiber sensor demonstrates the highest linearity of all three nano-materials.

Another interesting work is presented in [43] where the sensitivity of microstructured fibers (optical fibers that contain air holes in their cladding which extend along the entire length of the fiber) to gaseous oxygen is evaluated by using an inorganic matrix composed of tetraethoxysilne (TEOS) or methyltriethoxysilane (MTES). On the basis of the experimental results, the main conclusion is that both large hole diameters and thick walls between the holes are important for achieving good sensitivity of these MSFs to oxygen [43].

**Figure 6:**
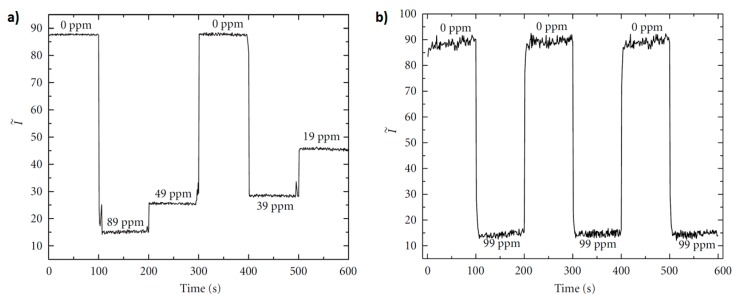
(**a**) Reversibility of the sensor response to DO changes; (**b**) Sensor response to switching from nitrogen-saturated to fully oxygenated environments. Reprinted with permission from Ref. [44].

A work based on crossed optical fiber sensor arrays for dissolved oxygen (DO) concentration measurements is presented in [44]. In this configuration, there is an “excitation fiber” that guides the excitation light and the luminescence is captured by a second fiber, the “detection fibers” which is placed at right angle to the “excitation fiber”. In this work, the Ru(II) complex is immobilized in a poly(ethylene glycol) (PEG) matrix which is attached covalently to the fiber surface in the cross junction. The experimental results related to the sensor reversibility as well as sensor response time can be shown in Figure 6. In both cases the referenced sensor intensity (I~) is plotted for varying oxygen concentrations. Upon switching from nitrogen-saturated to oxygen-saturated solution, I~ decreased with increasing DO concentration, as can be seen in Figure 6a. This process has been repeated several times with no significant change in I~ between the initial nitrogen-saturated solution and the final nitrogen-saturated solution, indicating a complete reversibility. In addition, the sensor response time by alternating successive oxygenation/deoxygenation cycles is observed in Figure 6b, being the calculated transition time of the order of 5–7 s, although it could be even shorter because the measured response time includes the time required to change between oxygenated and deoxygenated environments in the flow cell.

**Figure 7:**
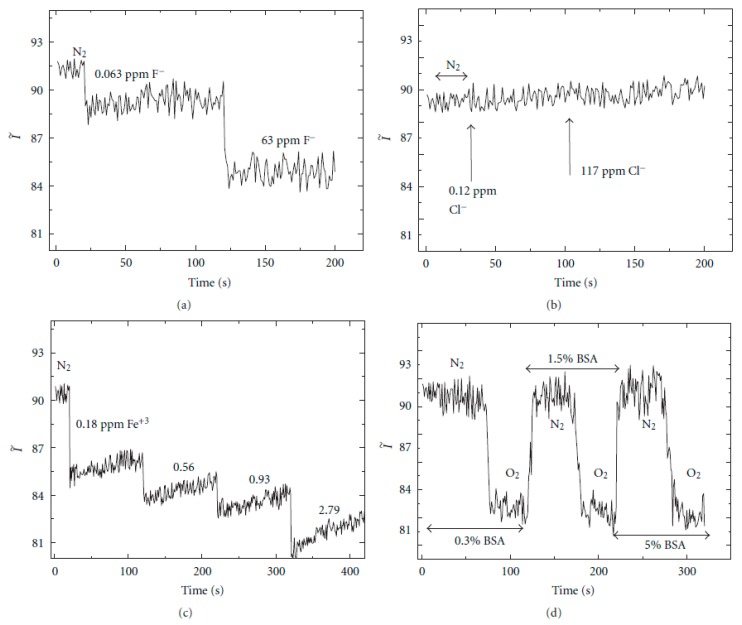
Potential interference of the DO measurement: sensor response to the presence of different species such as F^−^ (**a**), Cl^−^ (**b**), Fe^3+^ (**c**) and bovine serum albumin (BSA) (**d**). Reprinted with permission from Ref. [44].

An important consideration is that the matrix must protect the entrapped indicator from the environment, preventing interference with other agents. One of the research fields of a DO sensor is biological media (e.g., blood, rivers, food processing, etc.) where the contamination of the sensor surface with proteins and cells could affect the diffusion of oxygen into the sensor. According to this, the effects of nonspecific protein binding to the PEG-DA-film surface were investigated by the addition of bovine serum albumin (BSA, 0.3–5% (*w/v*) aqueous solution) to the flow-cell containing the fiber-fiber junctions immersed in deionized (DI) water, as it can be seen in Figure 7. First, the flow cell was purged with nitrogen, then, a 0.3% (*w/v*) BSA solution was added and allowed to interact with the fiber regions in the aqueous medium for 20 min. After this time, oxygen was introduced to the flow cell with a DO concentration of 1.5 ppm. As expected, the Ru complex intensity was quenched; after ∼75 min the sensor was flushed with nitrogen and the Ru complex intensity recovered to the initial values, indicating that no protein binding on the sensor surface occurred. The same trends have been observed with a higher BSA concentration of 5% (*w/v*), maintaining its recovery time and sensitivity. The results from the nonspecific protein-binding assay show the importance of encapsulating molecules inside the protective PEG-DA matrix, making this sensing material suitable for biological applications. However, this sensor shows vulnerability to metal ions (Fe^3+^) or notorious oxidizing species (F^−^), although the sensor response to these interferences is much smaller than the sensor response to oxygen.

Another fluorophore widely used for sensing applications is 1-hydroxypyrene-3,6,8-trisulfonate (HPTS) [46,47] which can be perfectly encapsulated by both sol-gel derived matrices [45,48,49,50] or polyelectrolyte multilayer structures [51,52,53]. In these works, applications such as carbon dioxide detection or pH sensing are studied. In works [48,49], the fluorescent dye is immobilized into hybrid silica matrices with the aim to create optical fiber carbon dioxide sensors. In both works, the main difference between them is the host silica matrix, showing a high sensitivity and good response time. 

**Figure 8:**
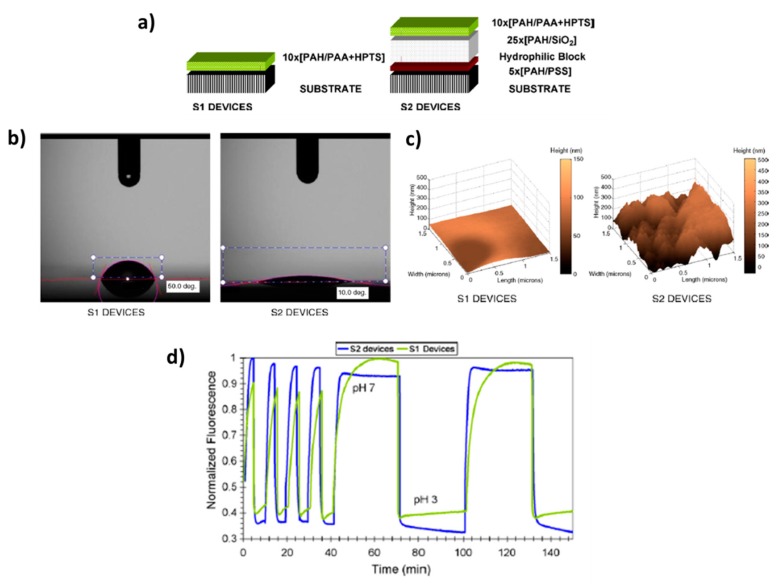
(**a**) Schematic representation of both structures (S1 and S2, respectively) for pH sensing; (**b**) Water contact angle (WCA) measurements and Atomic Force Microscopy (AFM) images (**c**) for S1 and S2, respectively; (**d**) normalized fluorescence emission responses of S1 and S2 when they are immersed in pH 3 and 7 buffers for 10 and 30 min of immersion time, respectively. Reprinted with permission of [53].

Another of the most interesting applications related to the use of HPTS is pH sensing [50,51,52,53]. An example of this can be found in [50] where the preparation of a fiber-optic sensing probe by using sol-gel process is presented and its employment for fluorescence detection of pH in small volumes of biosamples (droplets of plant xylen exudate). In work [52] several parameters are evaluated in order to minimize the photobleaching of the HPTS when it is encapsulated by a polymeric polyelectrolyte multilayer structure by using the Layer-by-Layer process. More specifically, three key fabrication parameters such as the composition of the fabrication atmosphere, a thermal curing process and the addition of an antifading agent to the polymeric matrix make possible an enhancement of the response time as well as a reduction of photobleaching effect. A similar work for obtaining an enhancement in the response time of pH sensing films can be found in [53] by means of using hydrophilic nanostructured coatings. In this work, two different LbL structures have been fabricated. The first one is just the “classical” polymeric sensitive coating with HPTS (named as S1) and the second one is composed of a high hydrophilic coating made of polymer and silica nanoparticles (SiNP) deposited prior to the sensitive one (names as S2). The experimental results obtained by water contact angle measurements of the samples corroborate this hydrophilic behavior which is associated to the higher roughness surface of the sample S2 introduced by the SiNP. This hydrophilic value added to the sensitive nanocoatings makes possible a better diffusion of water molecules and ions through the multilayer structure. Due to this, the response time of S2 sensor to pH changes between pH 3 to 7 is considerably lower than S1 sensors with an improvement of 5 times faster, from 15 min of the non-optimized device to 3 min. A summary of these experimental results is presented in Figure 8.

### 2.2. Immobilization of Metal Nanoparticles

**Figure 9:**
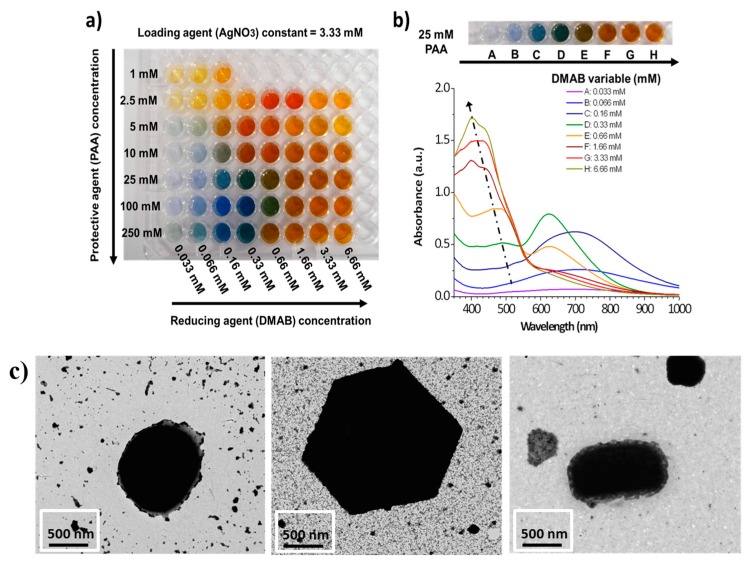
(**a**) Photograph of the multicolor silver map obtained as a function of variable protective agent (PAA from 1 to 250 mM) and reducing agent (DMAB from 0.033 to 6.66 mM), respectively; (**b**) UV-Vis spectra of the silver solutions prepared with variable DMAB concentration at a constant PAA concentration of 25 mM (fifth line of the multicolor silver map); (**c**) TEM images corresponding to AgNPs with spherical shape (orange color), hexagonal shape (green color) and rod shape (violet color), respectively. Reprinted with permission of [59,60].

As was mentioned before, the incorporation of nanoparticles can have a high impact on the sensing properties of the polymeric coatings and, in a complementary way, polymers also offer excellent approaches to deposit and immobilize nanoparticles. More specifically, metallic nanoparticles such as gold nanoparticles (AuNPs), silver nanoparticles (AgNPs), are of importance for sensing applications because of the presence of well-located strong absorption bands in both visible or infra-red regions, known as Localized Surface Plasmon Resonance (LSPR). LSPR is the result of the confinement of a surface plasmon in a nanoparticle smaller in comparable in size than the wavelength of the excitation light. This phenomenon can be also seen as a resonant coupling between the incident electromagnetic wave and the surface of the thin-film composed of the metallic nanoparticles. Part of the optical energy is transferred to the free electrons of the metal nanoparticles, resulting in an electromagnetic wave at the metal-dielectric interface and the apparition of sharp absorption peaks at a determined wavelength in the transmitted spectrum [54,55]. Multiple parameters such as shape, size, interparticle distance or aggregation state of the nanoparticles play a key role in the resultant wavelength position of the LSPR absorption bands [56,57,58,59]. A clear example is presented in Figure 9 where a multicolor silver map of 56 combinations is obtained as a function of a strict control of both protective and reducing agents during the synthesis process by using a chemical reduction method for a fixed molar concentration of the loading agent [59,60]. A wide range of AgNPs can be obtained with different colors (yellow, orange, red, violet, blue, green, brown) (Figure 9a) and the location of the LSPR absorption bands in the UV-Vis spectra is varied as a function of the resultant color (Figure 9b), indicating the synthesis of AgNPs with a tunable shape and size (Figure 9c), respectively.

**Figure 10:**
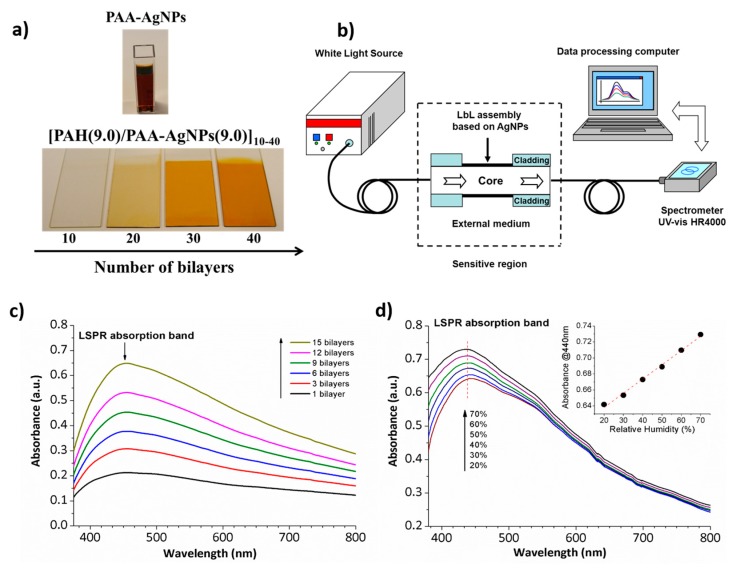
(**a**) Photograph of the PAA-AgNPs with an orange coloration and their incorporation into thin films by using the Layer-by-Layer assembly; (**b**) Experimental setup used to obtain and characterize the LSPR in the visible region; (**c**) UV-Vis spectra of the LbL coatings based on the incorporation of the AgNPs as a function of the thickness coating from 1 to 15 bilayers; (**d**) Spectral response of the LSPR-optical fiber sensor to RH changes from 20% to 70% RH. Reprinted with permission of [65].

One of the main benefits of using a polymeric protective agent such as Poly(acrylic acid, sodium salt) (PAA) for the synthesis of metallic nanoparticles is that this weak polyelectrolyte can be used to fabricate a multilayer buildup by using the LbL nano-assembly [61,62]. Once a good control over the synthesis of multicolor nanoparticles and the distribution of them in the resultant LbL films is obtained, the design of optical fiber sensors to detect physical parameters (refractive index, relative humidity) [63,64,65,66], chemical parameters (pH) [67,68] or biomedical parameters (human breathing) [69] is possible. A clear example can be found in Figure 10 where colored thin films based on the incorporation of metallic AgNPs with an orange coloration by using the LbL technique is presented (Figure 10a). In Figure 10b, the experimental setup used for the fabrication of polymeric sensitive LbL coatings onto an optical fiber core and for the characterization of the LSPR absorption band in the visible region inherent to the metallic nanoparticles. Figure 10c shows the UV-Vis spectra when the number of bilayers added in the final coating onto optical fiber core are increased from 1 to 15 bilayers. The spectra show a direct relation between the number of bilayers added (a higher thickness coating) and the increase of the absorbance value related to the LSPR absorption band at 440 nm. This is indicative that the amount of AgNPs incorporated into the LbL films directly increases with the thickness coating when the number of bilayers is gradually increased. In addition, the wavelength position of the LSPR band is maintained at 440 nm during the gradual incorporation of AgNPs, indicating a complete incorporation of spherical nanoparticles without any aggregation of them. Finally, Figure 10d shows the Relative Humidity (RH) response of the LSPR-optical fiber sensor where a change in intensity of the LSPR absorption band from 20% to 70% RH with a linear response (inset) is observed. 

**Figure 11:**
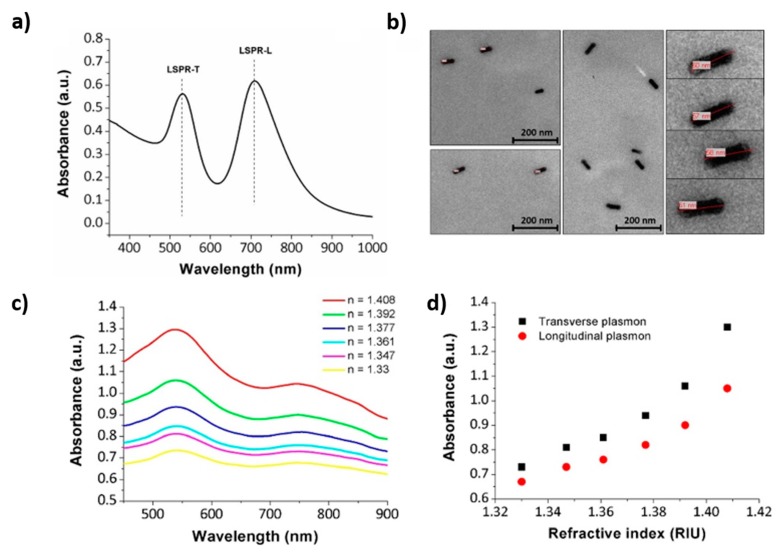
(**a**) UV-Vis absorption spectrum of the GNRs@PSS solution with two distinct absorption peaks at 530 nm (LSPR-T) and 710 nm (LSPR-L); (**b**) TEM micrographs and some zoom details of the synthesized GNRs; (**c**) Spectral response of the LSPR bands when the sensitive region is immersed in different Refractive Index (RI) solutions; (**d**) Growth in intensity of the LSPR-T and LSPR-L maxima to external RI variations. Reprinted with permission of [72].

Due to the high versatility of the LbL technique it has been also used for the immobilization of other types of metallic nanoparticles such as gold nanoparticles (AuNPs), even using different types of polyelectrolytes as protective agents. The presence of these AuNPs presents additional advantages such as biocompatibility, non-reactivity, good chemical stability, and easiness for a further functionalization [70,71]. The possibility of the implementation of self-assembled multilayers with metallic AuNPs adsorbed onto optical fibers provides a simple, fast, robust, and low-cost platform for LSPR sensing applications. In this sense, representative examples of the incorporation of metallic gold nanoparticles inside polymeric LbL coatings can be found in the bibliography for the design of LSPR optical fiber for sensing or biosensing purposes [72,73,74,75]. Works [72,73] show the incorporation of a specific type of gold nanoparticles with a well-defined shape into LbL films, known as nanorods (GNRs), for the design of optical fiber for measuring refractive index changes. These types of nanoparticles are of great interest because they offer strong plasmonic fields, providing two distinct plasmon resonance bands such as the transverse plasmon resonance band (LSPR-T) and the longitudinal plasmon resonance band (LSPR-L). A clear example can be found in Figure 11 where both absorption bands related to the LSPR phenomenon in the resultant UV-Vis spectra (Figure 11a) can be appreciated with their corresponding shape in the nanometric scale (Figure 11b). The optical response of the LSPR bands is presented in Figure 11c when the device is submitted to variable refractive index (RI) values. 

**Figure 12:**
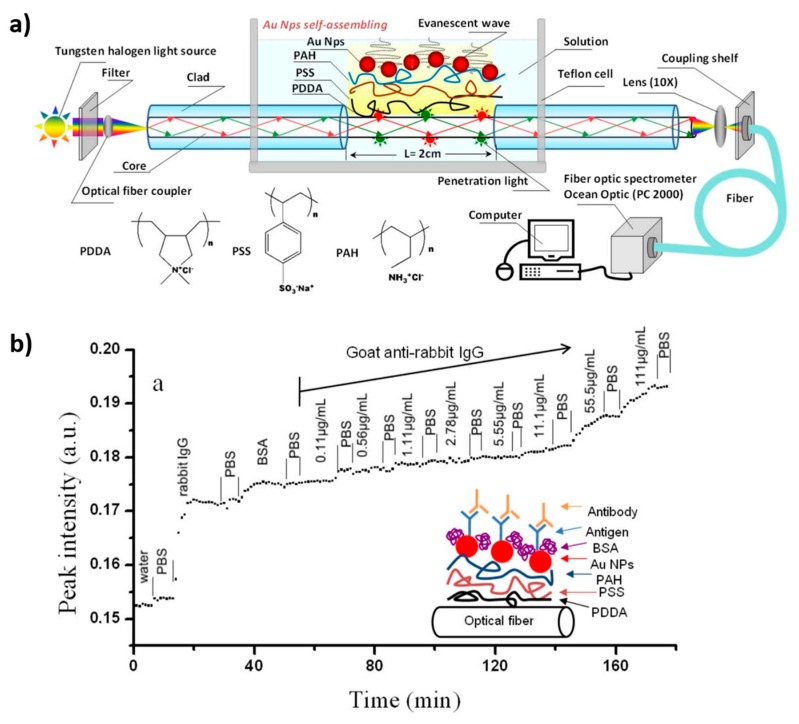
(**a**) Schematic representation of the experimental setup of the LSPR optical fiber sensor by using a trilayer polyelectrolyte structure which has been used to assemble the gold nanoparticles; (**b**) LSPR biosensing at different concentrations of goat anti-rabbit IgG from 0.11 to 100 μg/mL. Reprinted with permission from Ref. [74].

Another interesting work based on an optical fiber LSPR biosensor by gold nanoparticle assembly in a transmission setup architecture is presented in [74]. In this work, a gold nanoparticle assembled film is used as a sensing layer which has been built up onto a trilayer polyelectrolyte structure. The optical fiber LSPR sensor has been modified by rabbit IgG to detect goat anti-rabbit IgG, showing a high sensitivity and outstanding reproducibility. In Figure 12, it can be appreciated that the LSPR absorption band is gradually elevated when the goat anti-rabbit IgG concentration is increased. This response to an antibody makes it a promising alternative in biosensing applications as a portable immuno-sensor. 

A work is presented [75] which combines both SPR and LSPR for the detection of hydrogen peroxide. In this work, firstly the unclad portion of the fiber is coated with a silver layer by using thermal evaporation technique. In addition, secondly, this silver layer is coated with metallic silver nanoparticles which are embedded in a specific protective agent such as polyvinyl alcohol (PVA). A wavelength interrogation mode has been used to characterize the sensor, showing a resonance wavelength with the increase in the concentration of hydrogen peroxide. In addition, the resultant sensor shows a good selectivity because it presented negligible interference with other interfering agents (glucose, ethanol, or acetic acid).

Another aspect to note is that, among the fiber optic geometries in transmission architecture, U-shaped fiber optic probes have shown an important potential due to their high sensitivity, showing an enhancement in comparison with straight probes. According to this, the possibility of combining surface density of the metallic nanoparticles bound to U-bent probes makes possible the design of LSPR optical fiber sensors [76,77,78,79]. Work [76] evaluates the effect of both bend diameter and probe length in the resultant sensitivity of LSPR sensor to refractive index changes. Work [77] presents a comprehensive study about the plasmon penetration depth of an LSPR-based fiber optic biosensor. In this work, firstly two different methods to determine the LSPR penetration depth (*d*_p_) of the biosensor have been evaluated. In addition, secondly, the size of the gold nanoparticles (GNP) in the resultant penetration depth is evaluated. These results are of interest because the LSPR biosensor can be tailored to the biological analyte of interest such as small proteins as well as larger analytes (virus). Another interesting work can be found in [78] where the development of LSPR-based U-bent plastic optical fiber sensor is presented for the determination of refractive index. The experimental results indicate that the sensitivity is at the maximum when the bend diameter of the probes was varied from 2 to 3 times the fiber diameter. In [79] a U-bent fiber-optic volatile liquid sensor (acetone, methanol, ethanol and propanol, respectively) is presented based on the immobilization of noble nanoparticles (Ag and AuNPs) of an average diameter of 20 nm. In both cases, the response of the biosensors towards all volatile vapors shows a similar trend (sigmoid) with a quick response, the response being sensitivity for Ag nanoparticle coated probe more uniform than Au nanoparticle coated probe. 

**Figure 13:**
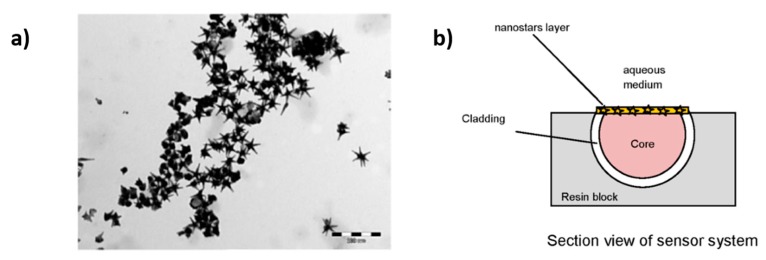
(**a**) TEM image of the five-branched gold nanostars (GNS); (**b**) Optical sensor system based on LSPR in POF. Reprinted with permission from Ref. [81].

It is important to note that the design of LSPR optical fiber sensors based on reflection architecture [80,81,82,83] can be also found in the bibliography. A representative example is presented in [80] for the detection of biotin-streptavidin bioconjugate pair where citrate-anion colloid AuNPs are used as polyanion and are deposited onto an optical fiber by using poly(allylamine hydrochloride) (PAH) as a polycation. The results indicate a wavelength shift related to the LSPR band as a function of the streptavidin concentration, showing a good sensitivity of 800 pg/mm^2^. In [81] a refractive index sensor is presented based on LSPR in a Plastic Optical Fiber (POF) by using five-branched gold nanostars (GNS) in a seed-growth synthesis with Triton X-100. In Figure 13, the aspect/ratio of the resultant nanoparticles when they are immobilized onto the POF can be observed, showing a sensitivity of 84 nm/RIU.

In another work, gold nanospheres have been immobilized onto the tip of an optical fiber with the aim to design a LSPR DNA biosensor [82]. A wavelength interrogation mode has been used to determine the sensitivity of the biosensor, showing a red shift of the LSPR band when the concentration is increased (from 0 to 500 nmol/L, respectively). 

Another work that combines the sol-gel technique for the immobilization of a specific type of nanoparticles such as palladium nanoparticles and a template of Triton X-100 is presented in [83] for the detection hydrogen gas. In this work, the agglomeration of the nanoparticles is prevented by using a porous support matrix, making possible that the nanoclusters are trapped within the micelle pores. In this sense and according to the use of sol-gel precursors, other works can be found in the bibliography for the immobilization of the nanoparticles [84,85,86,87,88]. A work is found in [84] where a fiber-optic probe based on a reflection mode combined with the immobilization of AuNPs into a sol-gel matrix is presented to quantify the hydrofluoric acid concentration in aqueous solution, it being possible to determine a relationship between HF concentration and silica sol-gel layer etching reduction. In works [85,86,87,88], the optical fiber has been silanized with a sol-gel precursor for a further immobilization of a specific type of gold nanoparticles. In [85], a LSPR optical fiber sensor is presented based on hollow gold nanostructures and it has been demonstrated that the resultant nanocages-sensor shows a significant improvement in the sensitivity to refractive index measurements. In [86], a comparative study by using solid gold nanoparticles (nanospheres and nanorods, respectively) is presented for the fabrication of LSPR-based optical fiber biosensors, showing in both cases the same detection limit of 1.6 nM to anti-human IgG. Other interesting work is found in [87] where the use of two different types of noble metal nanoparticles such as gold nanocages (AuNC) and gold nanospheres (AuNS) are evaluated for the design of protein optical fiber biosensor. It has been experimentally corroborated that by binding the noble metal nanoparticles has decreased the minimum detected target concentrations from 90 nM (reference sensor), 11 pM (AuNS) and 8 pM (AuNC), respectively.

Work [88] presents a novel work based on the immobilization of AuNPs which are encapsulated by graphene oxide (GO) in order to prevent the agglomeration of the metallic nanoparticles. The GO encapsulated AuNPs have been immobilized on the optical fiber core by a silanization process by using a sol-gel precursor to develop an optic sucrose sensor as a variation of the absorbance intensity peak of the LSPR absorption band. 

**Figure 14:**
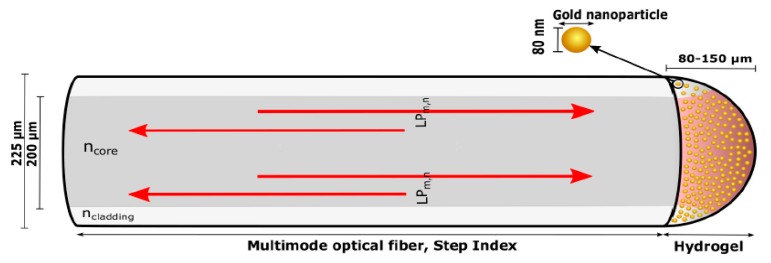
Illustration of the multimode optical fiber with hydrogel containing gold nanoparticles immobilized on a fiber end. Visible light is guided in the fiber core with the numerical aperture colored with red in the hydrogel. Reprinted with permission from Ref. [89].

Another different approach for the immobilization of the gold nanoparticles is using a stimuli-responsive hydrogel material which can be employed for measuring volumetric changes or well the binding to receptors on the nanoparticles surface [89,90,91]. A schematic representation of this stimuli responsive hydrogel can be found in Figure 14. In this Figure, a semi-spherical hydrogel containing nanoparticles with a spherical shape is deposited onto optical fiber end face. This configuration ensures a strong LSPR signal because of the high numerical aperture exciting a large fraction of the AuNPs and effective collection of scattering from the LSPR of the AuNPs [89]. This work evaluates the LSPR coupling and distribution of inter-particle distances between nanoparticles in the hydrogel on optical fiber end face as a function of polymer density of hydrogel which is controlled by a swelling/deswelling of the induced by pH changes. In works [90,91] is also used this same methodology for the design of multi-parameter fiber optic sensors. Work [90] demonstrates LSPR sensing of refractive index in the visible range and interferometric measurements of volumetric changes of the pH stimuli-responsive hydrogel in the infrared region. Work [91] demonstrates that AuNPs exhibit local surface plasmon resonance that is sensitive towards the refractive index of the surrounding medium, whereas the stimuli-responsive hydrogel is sensitive towards specific chemical compounds (ethanol solutions).

### 2.3. Immobilization of Metal Oxide Nanoparticles

**Figure 15:**
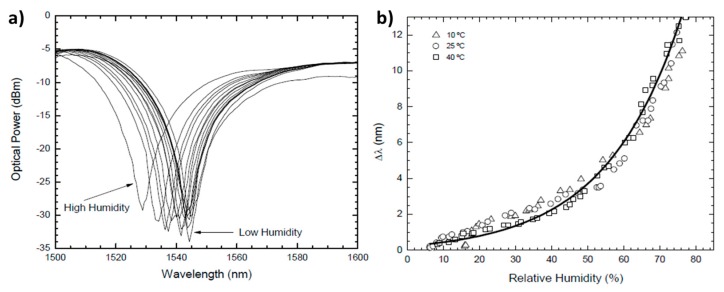
(**a**) Experimental results for different relative humidity levels; (**b**) Relative resonance wavelength shift with relative humidity levels for different temperature values. Reprinted with permission from Ref. [92].

As it was advanced, not only metal nanoparticles but also metal oxide nanoparticles such silica (SiO_2_) [92,93,94] or titania (TiO_2_) [95,96,97] can be used for sensing applications. Metal oxide nanoparticles are very promising for sensing because they are mechanically and thermally robust, their properties are not affected by aging and show a good resistance to chemical degradation. Work [92] presents a humidity sensor based on a long-period fiber grating coated with a SiO_2_-nanospheres film by LbL nanoassembly. The experimental results indicate an exponential dependence of the resonance wavelength shift with humidity not dependent on the temperature, as it can be observed in Figure 15. 

Other interesting works based on the incorporation of SiO_2_ nanoparticles into a polymeric thin film for humidity sensing on wounds are presented in [93,94]. This type of optical fiber sensors can be incorporated into wound dressings to monitor moisture and predict the healing without the need of removing the dressing. In these works, the Layer-by-Layer technique has been used to coat the central unclad section of a multimode polymeric optical fiber to fabricate a hydrophilic film by using a polycation and SiO_2_ mesoporous nanoparticles. The sensor shows a decrease in light transmission as relativity humidity increases because of refractive index changes of the coating. In addition, these experimental results provide a faster response to changes in the humidity of the skin microenvironments than a commercial sensor. 

Other works that also used the LbL technique for the incorporation of other different types of metal oxide nanoparticles such as TiO_2_ nanoparticles into a polymeric thin film can be found in [95,96,97] for different sensing applications (humidity or refractive index sensors). In these works, a new optical phenomenon is presented known as lossy-mode resonance (LMR) [98], which presents additional advantages in comparison with LSPR phenomenon. One of these advantages is that LMR can be generated by using a broader range of supporting materials, not only electrically conductive materials as in the LSPR-devices. In addition, another important advantage is that the LMR-devices are generated by both, TE and TM, light polarizations. More details about this optical phenomenon will be commented in the following section when the polymeric matrix is used as a selective element for sensing applications. 

**Figure 16:**
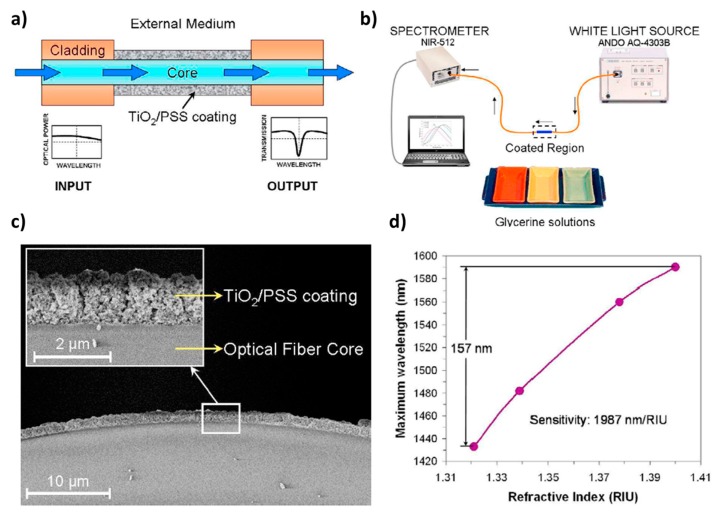
(**a**) Schematic representation of the optical fiber device; (**b**) Optical fiber transmission setup used to characterize the refractometer response; (**c**) SEM image and detail of the coated optical fiber core; (**d**) Variation of the maximum absorbance wavelength if the refractive index of the external medium is changed from 1.32 to 1.40. Reprinted with permission from Ref. [97].

Finally, according to this specific optical phenomenon, an optical fiber refractometer based on LMR supported by a TiO_2_ coating is presented in Figure 16. In this figure, the LbL method has been applied to fabricate a coating of TiO_2_ nanoparticles onto the multimode optical fiber core [99]. This device shows an absorption peak in the infrared region which shifts to higher wavelengths when the refractive index of the surrounding medium becomes higher. The sensitivity of this refractometer is 1987 nm/RIU, reaching a dynamical range of 157 nm when the external refractive index varies between 1.32 and 1.40.

## 3. Active Polymers for Optical Fiber Sensors

In this section, polymers play an active role in the sensing mechanism, rather than acting as simple support matrix for the active elements. There are many different approaches in which polymers can interact with light and be used as sensitive element in optical fiber sensors. For example, the variation of the effective refractive index of the polymeric film can affect to the evanescent losses of the optical fiber, or create optical interferences, or even can affect to some optical resonances. The active polymer element can modify its morphology (thickness, roughness), or its optical properties due to different phenomena such as swelling, piezoelectric behavior, or even selective adsorption of small molecules. Therefore, it is very common that a family of polymers, which have a sensitivity to a certain physical, chemical, or biological magnitude, can be used in several optical arrangements and devices, making possible different optical fiber sensors.

**Figure 17:**
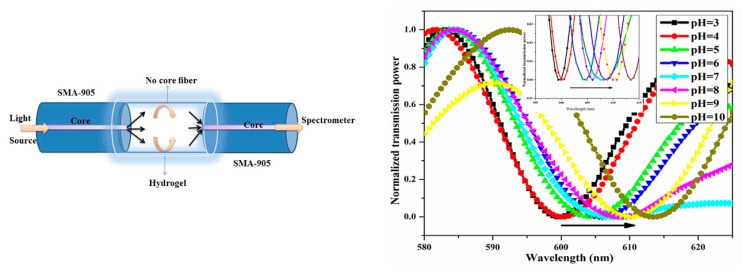
Hydrogel-based pH optical fiber interferometric sensor. (**Left**) Schematic representation of the optical fiber device. The NCF segment acts as interferometric cavity. (**Right**) Optical spectral response of the sensor when immersed into different pH solutions. The minima wavelength shows a direct relationship with the pH of the solution. Reprinted with permission from Ref. [101].

For example, hydrogels have been very used to create humidity and pH optical fiber sensors. Hydrogels are formed by networks of crosslinked hydrophilic polymer chains that form a tridimensional structure with a high flexibility, and the ability to absorb a high amount of water. This highly hydrophilic characteristic has been used to create evanescent wave optical fiber sensors [100]. In this work, the authors use Plastic Cladding Fibers (PCF) to create the sensors. A segment of 5 cm of cladding has been mechanically removed and the exposed core has been coated with an agarose hydrogel thick coating. The change in the effective refractive index of the hydrogel layer as it absorbed environmental humidity had a direct impact on the evanescent losses, reducing the amount of transmitted light as Relative Humidity (RH) decreases. In addition, hydrogels are also sensitive to environmental pH variations, showing significant morphological alterations due to pH changes of their surrounding medium. Consequently, it is possible to find different approaches with similar hydrogels for pH sensing applications [101]. In this case, two Single Mode optical fibers (SMF) have been spliced to a No-Core Optical Fiber (NCF), creating an interferometric cavity, and the NCF segment has been coated with a layer of agarose hydrogel (see Figure 17). 

Other authors have used the same principle with different optical structures, such as optical fiber gratings, more specifically, Long-Period Gratings (LPGs). These LPG are optical fibers in which a periodic perturbation of the refractive index of the core has been created, with a long period (hundreds of microns) with respect to the standard Fiber Bragg Gratings. This periodical perturbation creates an optical resonance with certain propagating wavelengths that are guided through the cladding of the LPG. This resonance condition is intrinsically sensitive to temperature and strain, as far as both parameters may cause modifications on the grating period, but it is also sensitive to the effective refractive index of the cladding surrounding medium. As the external hydrogel coating modifies its optical properties induced by relative humidity, the resonance wavelength of the LPG cladding modes is also modified [102]. Other authors have reported the use of modified polyacryamide hydrogels using aminophenylboronic acid and cationic dimethylaminopropyl acrylamide (DMAPAA) to measure the amount of several polysacharides, and mores specifically glucose in similar experimental conditions as human blood (pH, ionic strength, etc.) [103,104]. 

**Figure 18:**
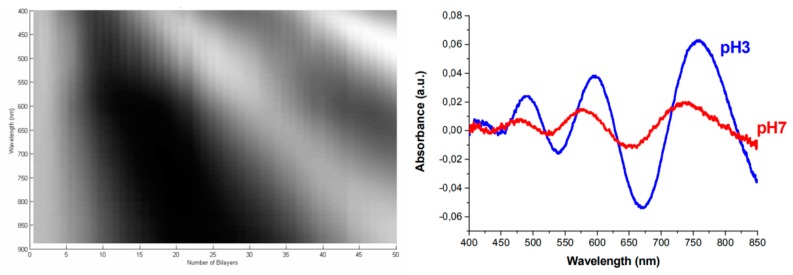
Low coherence white light interferometric Fabry-Perot cavity created using (PAH/PAA) LbL thin films. (**Left**) Evolution of the interference pattern as the number of bilayers is increased, using a halogen lamp as light source. (**Right**) Spectral response of the nano-FP cavity when the optical fiber tip is immersed into different pH solutions. Reprinted with permission from Ref. [105].

Nevertheless, it is not just classic hydrogels that are suitable for this kind of application. Layer-by-Layer (LbL) polymeric assemblies can be used for humidity and pH sensing. Such LbL coatings offer additional advantages since it is possible to keep a very good control of the composition and morphological characteristics of the films independently from the geometry of the substrate. The good control of the LbL sensitive films can be used to create ultra-thin interferometric cavities directly at the end face of perpendicularly cleaved optical fibers [105]. Figure 18 shows the optical response of the sensor as the (PAH/PAA) LbL multilayer is sequentially built up to 50 bilayers. The high quality and the low thickness of the (PAH/PAA) coatings induces the formation of a Fabry-Perot cavity that shows a classical interference pattern even with low-coherence light sources such as a halogen lamp. Since PAH and PAA are weak polyelectrolytes, the morphological properties of the polymeric chains are strongly pH-dependent, and these pH changes may induce significant changes in the thickness, roughness and effective refractive index of the LbL thin-film [62]. The interferometric characteristic of such sensors offers additional advantages respect to the previous evanescent approaches, as far as the interferometric responses are wavelength-based signals, which means that the measurement will be robust against intensity fluctuations of the light.

Under certain conditions the polymeric overlays can modify substantially the guiding conditions of the optical fibers, creating interesting optical resonances. As has been previously mentioned in Section 2.3, certain overlays can create some resonant overlay-guided modes, called Lossy Mode Resonances (LMR), and such phenomenon is very useful in sensors applications due to their wavelength-based measuring signals, and their extremely high sensitivity [106,107]. In some works, an overlay of ITO, or Sn_2_O_3_, is firstly created onto the optical fiber core, and on top of that an external overlay is fabricated using a sensitive polymer [98]. The LMR resonances created into the first overlay are strongly affected by the effective refractive index of its surrounding medium, consequently any change in the external polymeric overlay will have a significant effect in the resonant conditions [108]. In some cases, the presence of the first ITO LMR-supporting layer is not necessary, and the sensitive polymeric coating can create the LMR phenomenon on its own [15].

**Figure 19:**
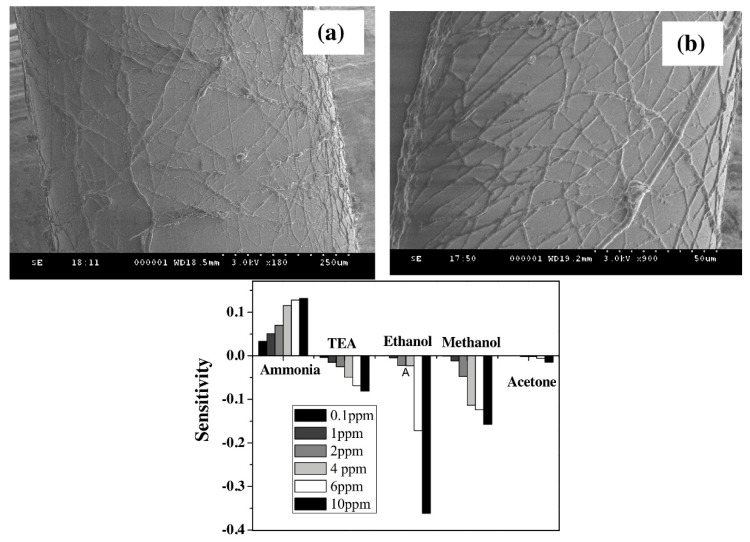
Electrospun nanofibers PPy/PEO. (**Top**) (**a**,**b**) SEM images of the electrospun fibers over the core of the 600 µm optical fiber. (**Bottom**) Sensitivity of PPy/PEO coated optical fiber against ammonia, triethylamine, ethanol, methanol, and acetone vapors at different concentrations. Reprinted with permission from Ref. [109].

Other works have used polymeric micro and nano-fibers fabricated by electrospinning. With this technique it is possible to create fibrous membranes for many applications such as filtering, wound dressing, etc. The high specific surface of such nano-webs is very attractive for the sensor field since it could lead to faster and more sensitive devices. For example, there are works in which electrospun nanofibers of polypyrrole and polyethylene oxide (PPy/PEO) are projected over the surface of a 600 µm core optical fiber and then they are exposed to Volatile Organic Compounds (VOCs) such as ammonia, trimethylamine, ethanol, and methanol in the ppm concentration range [109]. The resultant PPy/PEO microfibers are shown in Figure 19, and have an approximate diameter of 2 µm. 

Other polymers can be found in electrospinning applications for sensors, for example the hydrophilic polymer Poly(acrylic acid) has been used as a relative humidity sensitive overlay [110], showing very fast response times and making possible its use as a human breathing monitor. Alternatively, Poly(vinylidene fluoride) (PVDF) has been also used to create fiber mats for humidity sensing [111]. A similar PAA microfiber overlay has been used to detect pH variations in aqueous media, showing sensitivities around 0.53 dB/pH unit [112]. 

Additionally, there are other optical arrangements in which polymeric thin films can be used to create optical fiber sensors. For example, the Surface Plasmon Resonance (SPR) phenomenon, typically studied using the Kretschmann configuration, can be also implemented into optical fiber setups to reproduce the SPR phenomenon [113,114,115]. Although it is possible to find numerous works which use the SPR arrangement for creating optical fiber sensors, many of them are based on the adsorption of the analytes directly to the functionalized surface of the conductive SPR layer, without involving any polymer. 

**Figure 20:**
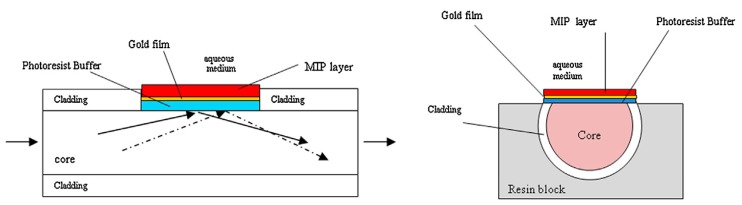
Plastic optical fiber SPR-based TNT sensor. (**Left)** Longitudinal-sectional diagram of the sensor. It depicted the gold thin film for the SPR generation, and the MIP external TNT sensitive layer. (**Right**) Cross-section view of the sensor showing the side-polished sensitive region. Reprinted with permission from Ref. [122].

Nevertheless, Molecular Imprinted Polymers (MIPs) are a clear example of polymer engineering success to achieve an innovative non-biological material that can selectively adsorb small molecules. The combination of classical optical fiber SPR sensors with MIPs is a hot topic in the scientific community since it makes possible to obtain highly sensitive and selective sensors. There are many examples of SPR-based optical sensors that uses MIP technology to create their sensitive polymeric layers [116,117,118,119,120,121]. For example, Cennamo and co-workers have reported a MIPs-based SPR optical fiber sensor to detect Trinitrotoluene (TNT) [122] (see Figure 20). This sensor allows the direct detection of a low molecular mass substance even at low concentration, which always have been very problematic for any kind of sensor. Thanks to the high sensitivity of the SPR phenomenon it is possible to detect the small refractive index changes in the MIP overlay as the target molecule (TNT) was selectively adsorbed by the MIP. This is especially remarkable since this sensor do not involve the use of any electrical devices in particularly critical conditions, as the presence of explosive materials.

## 4. Final Remarks

The possibility of using optical fibers functionalized with chemically sensitive polymeric layers can be employed as an interesting platform for the development of sensing or biosensing applications. A clear example is the use of optical fiber sensors to improve the functionality of medical devices for biomarker detection or drug monitoring because sensitive materials can exhibit changes in their optical properties upon the exposure to an analyte of interest, showing a very fast response with a high degree of sensitivity and selectivity. Finally, due to the high versatility of the optical fiber combined with polymeric layers making possible their implementation in other fields for the detection of chemical as well as physical parameters (pH, refractive index, relative humidity, gases, heavy metals), it is necessary to have a good control over these parameters in industrial processes. 

## 5. Conclusions

This review is focused on the utilization of polymeric coatings on optical fibers for sensing or biosensing applications. It is well known that optical fiber technology presents several advantages such as the absence of electromagnetic interferences, high degree of miniaturization or capability of remote sensing and multiplexing that other technologies are unable to offer, being an ideal candidate for a wide variety of sensing purposes. The combination of the intrinsic properties related to optical fibers with polymeric sensitive coatings makes possible the fabrication of optical fiber devices with an important enhancement in key factors for sensing applications such as selectivity, sensitivity, response time or even cross-reference measurements. In this work, two different roles which polymers can play for potential sensing applications are evaluated. In the first one, the polymeric matrix is used as a solid support for the immobilization of a specific chemical transducer (luminescent materials, metallic or metal oxide nanoparticles). In the second one, the polymeric matrix is used directly as a chemical transducer (hydrogels, weak-polyelectrolyte thin films, electrospun nanofibers or MIPs). Finally, in both cases, as a solid support for active polymers, potential applications of the resultant optical fiber sensors in fields as diverse as biology, chemistry, engineering, industry, or medicine are presented.
